# Prognostic accuracy of 70 individual frailty biomarkers in predicting mortality in the Canadian Longitudinal Study on Aging

**DOI:** 10.1007/s11357-023-01055-2

**Published:** 2024-01-06

**Authors:** Joanna M. Blodgett, Mario Ulisses Pérez-Zepeda, Judith Godin, Dustin Scott Kehler, Melissa K. Andrew, Susan Kirkland, Kenneth Rockwood, Olga Theou

**Affiliations:** 1 Division of Geriatric Medicine, Dalhousie University and Nova Scotia Health, Halifax, NS Canada; 2 Division of Surgery Interventional Science, Institute of Sport Exercise and Health, University College London, London, UK; 3 Instituto Nacional de Geriatría, Mexico City, Mexico; 4 Centro de Investigación en Ciencias de La Salud (CICSA), FCS, Universidad Anáhuac México Campus Norte, Huixquilucan, Edo. de México, Lomas Anahuac Mexico; 5 School of Physiotherapy, Dalhousie University, Halifax, NS Canada; 6 Department of Community Health and Epidemiology, Dalhousie University, Halifax, NS Canada

**Keywords:** Frailty, Biomarkers, Prediction, Mortality, CLSA

## Abstract

The frailty index (FI) uses a deficit accumulation approach to derive a single, comprehensive, and replicable indicator of age-related health status. Yet, many researchers continue to seek a single “frailty biomarker” to facilitate clinical screening. We investigated the prognostic accuracy of 70 individual biomarkers in predicting mortality, comparing each with a composite FI. A total of 29,341 individuals from the comprehensive cohort of the Canadian Longitudinal Study on Aging were included (mean, 59.4 ± 9.9 years; 50.3% female). Twenty-three blood-based biomarkers and 47 test-based biomarkers (e.g., physical, cardiac, cardiology) were examined. Two composite FIs were derived: FI-Blood and FI-Examination. Mortality status was ascertained using provincial vital statistics linkages and contact with next of kin. Areas under the curve were calculated to compare prognostic accuracy across models (i.e., age, sex, biomarker, FI) in predicting mortality. Compared to an age-sex only model, the addition of individual biomarkers demonstrated improved model fit for 24/70 biomarkers (11 blood, 13 test-based). Inclusion of FI-Blood or FI-Examination improved mortality prediction when compared to any of the 70 biomarker-age-sex models. Individual addition of seven biomarkers (walking speed, chair rise, time up and go, pulse, red blood cell distribution width, C-reactive protein, white blood cells) demonstrated an improved fit when added to the age-sex-FI model. FI scores had better mortality risk prediction than any biomarker. Although seven biomarkers demonstrated improved prognostic accuracy when considered alongside an FI score, all biomarkers had worse prognostic accuracy on their own. Rather than a single biomarker test, implementation of routine FI assessment in clinical settings may provide a more accurate and reliable screening tool to identify those at increased risk of adverse outcomes.

**Supplementary Information:**

The online version contains supplementary material available at 10.1007/s11357-023-01055-2.

## Introduction

The global population has aged rapidly in recent decades [1]. In Canada, the proportion of individuals aged 65 + or older is expected to double from 2018 to 2030 [2]. This changing age demographic and implications for health and mortality outcomes are crucial to help manage the complexities of population aging at an individual, society, and governmental level. One quantifiable approach to aging considers frailty as “a state of increased vulnerability to poor resolution of homeostasis, which increases the risk of adverse outcomes, including falls, delirium, and disability” [3]. This vulnerability usually represents age-related decline across multiple physiological systems and is commonly operationalized using a frailty index (FI). An FI can quantify the state of any individual’s health as the proportion of health variables that are considered to be in a deficit state [4]. Across heterogenous samples and settings, FIs demonstrate replicable properties that are consistent regardless of the individual deficits included.

Most commonly used in large cohort studies, FIs are increasingly employed in clinical settings where they can be derived from routine administrative data [5, 6], Comprehensive Geriatric Assessments [7], or existing medical records. For example, when electronic frailty index (eFIs) derived from routinely collected medical records have been implemented internationally, they show a strong discriminative capacity for predicting mortality, hospitalization, and other adverse outcomes [5, 8–15]. This success demonstrates the substantial potential and feasibility of automated frailty screening in primary and secondary health settings. Still, many researchers aim to simplify the FI approach by substituting a single “frailty biomarker” in the hope of parsimoniously predicting adverse outcomes. To date, such attempts have not been successful [16–19]. To explore whether any single biomarker might do well enough on its own to supplant combinations of biomarkers, our aim was to investigate and compare the prognostic accuracy of 70 individual biomarkers in predicting mortality with previously validated blood- and examination-based frailty indices [20].

## Methods

### Sample

The Canadian Longitudinal Study on Aging (CLSA) is a study of community-dwelling older adults aged 45 to 85 at baseline (2010–2015). We used data from the baseline comprehensive cohort (*n* = 30,097), which measured clinical, biological, and physical assessments during a home or data collection site (DCS) visit. To be eligible for the comprehensive cohort, participants must live within 50 km of one of 11 DCSs across seven Canadian provinces. Detailed information on the CLSA objectives, sampling strategy, protocol, and sample characteristics is available elsewhere [21].

### Frailty biomarkers

A total of 70 frailty biomarkers were measured including 23 blood biomarkers from non-fasting blood samples (i.e., triglycerides, hematocrit, albumin; see Table 1 for a full list) and 47 test-based biomarkers. Test-based biomarkers consisted of 5 physical performance measures, 9 cognitive tests, 7 anthropometric measures, 2 spirometry measures, 9 hearing or vision measures, and 15 cardiac indicators (see Table 2 for a full list).

**Table 1:** Blood-based biomarkers and their impact on mortality prediction based on comparisons of area under the curve (AUC) across four logistic regression models

	Biomarker improved age + sex model (Model 2 vs 1)	FI improved age + sex model (Model 3 vs 1)	FI improved age, sex + biomarker model (Model 4 vs 2)	Biomarker improved age, sex + FI model (Model 4 vs 3)
Red blood cell distribution width	✓	✓	✓	✓
High-sensitivity C-reactive protein	✓	✓	✓	✓
White blood cells	✓	✓	✓	✓
Hemoglobin A1c	✓	✓	✓	–
Hematocrit	✓	✓	✓	–
Mean corpuscular hemoglobin	✓	✓	✓	–
Hemoglobin	✓	✓	✓	–
Red blood cells	✓	✓	✓	–
Albumin	✓	✓	✓	–
Creatinine	✓	✓	✓	–
Free thyroxine	✓	✓	✓	–
Mean corpuscular volume	–	✓	✓	–
Mean platelet volume	–	✓	✓	–
Cholesterol	–	✓	✓	–
Ferritin	–	✓	✓	–
Triglycerides	–	✓	✓	–
Granulocytes	–	✓	✓	–
Lymphocytes	–	✓	✓	–
Monocytes	–	✓	✓	–
Platelets	–	✓	✓	–
25-Hydroxyvitamin D	–	✓	✓	–
Estimated glomerular filtration rate	–	✓	✓	–
Thyroid-stimulating hormone	–	✓	✓	–
Total	**11**	**23**	**23**	**3**

Model 1: age and sex; Model 2: age, sex, biomarker; Model 3: age, sex, 22-item FI; Model 4: age, sex, biomarker, 22-item FI

✓: improved prognostic accuracy (statistically significant improvement in AUC between models using Benjamini–Hochberg correction with false discovery rate of 0.05)

–: no statistically significant difference in prognostic accuracy

**Table 2:** Test-based biomarkers and their impact on mortality prediction based on comparison of area under the curve across four models

	Biomarker improved age + sex model (Model 2 vs 1)	FI improved age + sex model (Model 3 vs 1)	FI improved age, sex + biomarker model (Model 4 vs 2)	Biomarker improved age, sex + FI model (Model 4 vs 3)
Physical performance measures				
Timed 4-m walk	✓	✓	✓	✓
Chair rise	✓	✓	✓	✓
Timed get up and go	✓	✓	✓	✓
Standing balance	✓	✓	✓	–
Grip strength	✓	✓	✓	–
Cognitive measures				
Stroop interference time	✓	✓	✓	–
Delayed recall	✓	✓	✓	–
Event-based memory	✓	✓	✓	–
Animal fluency	–	✓	✓	–
Controlled oral word association	–	✓	✓	–
Immediate recall	–	✓	✓	–
Mental alteration test	–	✓	✓	–
Choice reaction time	–	✓	✓	–
Time-based memory	–	✓	✓	–
Anthropometric measures				
Waist-hip ratio	✓	✓	✓	–
Body mass index	–	✓	✓	–
Whole body BMD, T-score	–	✓	✓	–
BMD, multiple body regions	–	✓	✓	–
Appendage lean mass	–	✓	✓	–
Body fat percent	–	✓	✓	–
Adiposity, multiple body regions	–	✓	✓	–
Spirometry measures				
Forced vital capacity (FVC)	✓	✓	✓	–
FEV 1/FVC ratio	–	✓	✓	–
Hearing and vision measures				
Hearing pure tone average, right	–	✓	✓	–
Hearing pure tone average, left	–	✓	✓	–
Visual acuity, left eye	–	✓	✓	–
Visual acuity, right eye	–	✓	✓	–
Intraocular pressure, right	–	✓	✓	–
Intraocular pressure, left	–	✓	✓	–
Corneal hysteresis, right	–	✓	✓	–
Corneal hysteresis, left	–	✓	✓	–
Mean ocular perfusion pressure	–	✓	✓	–
Cardiac measures				
Pulse	✓	✓	✓	✓
Max carotid intima thickness	✓	✓	✓	–
ECG, QT interval	✓	✓	✓	–
ECG, PQ interval	–	✓	✓	–
ECG, P axis	–	✓	✓	–
ECG, R axis	–	✓	✓	–
ECG, T axis	–	✓	✓	–
ECG, P duration	–	✓	✓	–
ECG, QRS duration	–	✓	✓	–
ECG diagnosis summary	–	✓	✓	–
Systolic BP	–	✓	✓	–
Diastolic BP	–	✓	✓	–
Pulse pressure	–	✓	✓	–
Carotid intima thickness, right	–	✓	✓	–
Carotid intima thickness, left	–	✓	✓	–
** Total**	**13**	**47**	**47**	**4**

Model 1: age and sex; Model 2: age, sex, biomarker; Model 3: age, sex, 22-item FI; Model 4: age, sex, biomarker, 22-item FI

✓: improved prognostic accuracy (statistically significant improvement in AUC between models using Benjamini–Hochberg correction with false discovery rate of 0.05);

–: no statistically significant difference in prognostic accuracy

### Frailty indices

Two FIs were constructed: an FI-Blood, consisting of the 23 blood biomarkers, and an FI-Examination, consisting of the 47 examination-based tests. Details of FI construction including a detailed data dictionary and syntax files have been previously documented [20]. Briefly, deficits were selected for inclusion following four standard criteria [22]: deficits must be health-related, increase with age, not saturate too early, and cover a range of health domains. Each deficit was coded on a scale from 0 (no deficit) to 1 (highest level of the deficit) using binary or ordinal cut-points or transformation into normalized scores. For example, abnormal blood tests such as albumin or hemoglobin were coded as 0 if the score fell within the normal range and 1 if the score fell outside. Variables such as physical performance and cognitive scores were normalized such that 0 indicated no deficit and 1 indicated the highest deficit level. An individual must have data on 80% of deficits for an FI score to be derived. FI scores were calculated as the sum of all deficits present divided by the number of deficits considered (e.g., 20 of 40 deficits = FI score of 0.5).

### Outcomes

Mortality status as of July 1, 2019, was ascertained using linkage to provincial vital statistics, contact with participants between waves of data collection, or direct contact from the next of kin. Censoring time was calculated as the time between the day of the data collection site visit (i.e., between 2010 and 2015) and July 1, 2019 (censoring date). The exact time to death is not currently available.

### Statistical analyses

For each individual frailty biomarker, area under the curves (AUCs) were calculated to examine the prognostic accuracy of four different logistic regression models in predicting mortality. The four models included the following predictors: (1) age and sex; (2) age, sex, biomarker; (3) age, sex, FI (22-item FI-Blood or 46-item FI-Examination); (4) age, sex, biomarker, FI. Note that in Models 3 and 4, the relevant biomarker was excluded from the FI to avoid collinearity.

We test the equality of AUCs and compare the prognostic accuracy of different models using *roccomp* package, which applies multiple test modalities to the sample. First, the addition of each biomarker to an age-sex model (Model 2) was compared to the age-sex only model (Model 1), followed by a comparison of the age-sex-FI model (Model 3) to the age-sex only model. Next, the addition of FI score to the age-sex-individual biomarker model was examined (Model 4 vs Model 2). Finally, the reverse was considered, which examined the addition of the individual biomarker to the age-sex-FI model (Model 4 vs Model 3). Due to some participants having no blood tests, sample size varies between biomarkers. However, for each of the four models for any given biomarker, we restricted to the same sample size to enable direct comparison between AUCs. To control for multiple comparisons, the Benjamini–Hochberg correction was used to determine statistical significance [23], and a false discovery rate of 0.05 was used to calculate the critical values. Analytic weights were applied to all models to provide population-representative estimates [24]. Characteristics of those who survived and died as of mortality censoring data were compared. All analyses were conducted in Stata 16.

## Results

Of the 30,097 comprehensive cohort participants, FI-Examination data and FI-Blood were available for 29,341 and 25,253 individuals, respectively [20]. Of the maximal 29,341 participants included in analyses, the mean age was 59.4 (SD 9.9) years and 50.3% (*n* = 14,762) were female. As of July 2019, 899 (3.1%) had died. The average time between baseline data collection and mortality ascertainment was 5.5 ± 0.8 years (range, 4.0–7.6). Participants had a mean FI-Blood score of 0.15 ± 0.10 (range, 0.00–0.70) and a mean FI-Examination score of 0.27 ± 0.08 (range, 0.07–0.70). Supplementary File 1 describes baseline characteristics by mortality status; as expected, those who survived were younger (63 ± 10 vs 72 ± 9), were more likely to be female (51% vs 39%), had higher educational attainment (78% with post-secondary degree/diploma vs 66%), and had lower frailty scores (FI-Blood, 0.16 ± 0.19 vs0.24 ± 0.13; FI-Examination, 0.29 ± 0.08 vs 0.38 ± 0.10).

### Blood-based biomarkers

**Fig. 1:**
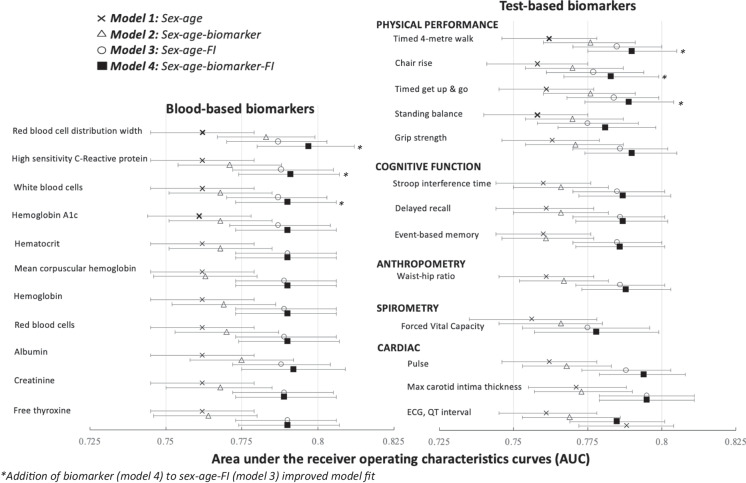
Area under the receiving operating characteristic (AUC) with 95% confidence intervals for the 11 blood-based and 13 test-based biomarkers that improved mortality prediction compared to a sex-age only model (model 2 vs model 1)

Table 1 provides a summary of model findings of AUC comparisons (95% confidence intervals) for each blood-based biomarker model, Fig. 1A highlights the biomarkers with notable findings and Supplemental File 2 provides complete model details. The addition of the individual biomarkers to the age-sex model (Model 2) improved model fit in 11 of 23 models. Conversely, the addition of FI-Blood (Model 3) improved the prognostic accuracy of the age-sex model in all models (range, 0.787 (0.770, 0.803) to 0.791 (0.775, 0.808); note that AUCs range due to fluctuations in sample size across biomarkers).

The addition of FI-Blood score (Model 4) to the age-sex-biomarker model (Model 2) also improved the prognostic accuracy for all 23 blood-based biomarkers. When the reverse was examined, the addition of individual biomarkers (Model 4) to the age-sex-FI model (Model 3) improved prognostic accuracy for only three biomarkers: red blood cell distribution width, high-sensitivity C-reactive protein, and white blood cells. Combining age, sex, FI-Blood, and red blood cell distribution width yielded the highest AUC (0.797 (0.781, 0.813)) of any model.

### Test-based biomarkers

As above, a summary of the comparison of all AUCs for the test-based biomarker models is provided in Table 2, Fig. 1B highlights the biomarkers with notable findings. Results were similar to that of the blood-based biomarkers and Supplemental File 3 provides complete model details. The addition of individual biomarkers (Model 2) demonstrated a better model fit than the base age-sex model in 13 of 47 models; this included all five physical performance measures, three cognitive measures, three cardiac measures, forced vital capacity, and waist-hip ratio. The addition of the FI-Examination (Model 3) improved the prognostic accuracy of the age-sex model for all biomarkers.

As with the blood-based biomarkers, the addition of FI-Examination (Model 4) to each of the 47 age-sex-biomarker models (Model 2) improved model fit. Conversely, adding the individual biomarker to the age-sex-FI model only improved model fit for four biomarkers: three physical performance measures (walking speed, chair rise, time up and go) and pulse. The largest AUC was produced for the age, sex, FI, and average carotid intima—right side model (0.798 (0.782, 0.814)).

## Discussion

In a large cohort of nearly 30,000 individuals aged 45 + , we demonstrated that no single biomarker provided sufficient discriminative capacity in predicting mortality. Conversely, FI scores combining biomarkers demonstrated better mortality risk prediction when compared to all 70 individual biomarkers. There was some evidence to suggest that certain biomarkers (walking speed, chair rise, time up and go, pulse, red blood cell distribution width, high-sensitivity C-reactive protein, and white blood cells) can improve prognostic accuracy when considered in addition to frailty, yet on their own, they had poorer predictive validity than the comprehensive FI score. These findings reaffirm that measuring a single biomarker is an insufficient screening tool, further supporting the need for more automated and holistic FI assessment in clinical settings.

No biomarker predicted better mortality by itself when compared to the whole FI. However, the identification of seven biomarkers that improved the accuracy of the models requires further consideration, given their close relationship to aging. First, it is unsurprising that half of the physical performance tests added value to the predictive model, given these have been shown repeatedly to predict mortality [25]. Physical performance and pulse are the result of a complex interaction between bodily systems, which thus may be indicative of damage across cellular, organ, and multi-system levels. Aging has been shown to decrease this complexity, which therefore may eventually lead to adverse outcomes [26]. Each of the three blood-based markers (red blood cell distribution width, high-sensitivity C-reactive protein, and white blood cells) may suggest distinct mechanisms in the aging process, which may explain their utility in a prediction model. For example, C-reactive protein is a well-known inflammatory marker related to aging and adverse outcomes and is involved in immunosenescence and inflammaging [27]. Regarding red blood cell distribution width, a recent study showed that the hemoglobin to red blood cell distribution width ratio is associated with frailty [28]. Moreover, it is also associated with cognitive impairment, even in patients without anemia [29], suggesting multiple pathways through which it can affect the aging process. Finally, although white blood cell count is variable from day to day, it may be indicative of consistent acute infections, chronic stresses, or toxic exposures (i.e., smoking, obesity) [30] that can increase mortality risk. It is noteworthy that only overall white blood cell count, and not specific type (i.e., granulocytes, lymphocytes, monocytes), improved prognostic accuracy.

The clinical meaningfulness of the AUC differences warrants discussion. In health care settings, many clinicians strive to gather as much information as possible from the patient. Having a composite measure of frailty, such as the FI, allows clinicians to focus interventions on the whole individual, moving away from the reductionist focus that a sole biomarker would provide. For example, if C-reactive protein was the only available marker, clinical suspicion could orient the assessment and the subsequent intervention to target inflammatory disorders or cardiovascular stress. This approach would fail to acknowledge that the patient could benefit more from other interventions (e.g., exercise) and, instead, orient treatment to intervene on the single abnormal biomarker. Many of the deficits included are already routinely collected in clinical settings (e.g., pulse, vision, blood tests) or can be derived from self-report questionnaires; therefore, it is feasible to implement our findings into clinical care. Given the non-invasive nature of FI data collection, the statistically significant improvements in AUC (e.g., 0.02–0.04) are likely to provide a clinical net benefit, particularly considering the population level benefits if frailty assessments become a part of routine data collection across all clinical settings [31, 32].

Key strengths of this study include the large sample size, objective ascertainment of 70 individual biomarkers, and comprehensive triangulation of mortality status using three methods. Limitations include the lack of availability of exact date of death, missing blood samples in ~ 10% of sample, and exclusion criteria of CLSA (those with cognitive impairment, full-time members of Canadian Armed Forces, those in long-term care institutions, and those living on reserves/other aboriginal settlements). Future research should replicate these analyses while examining other adverse health outcomes including biomarker-specific disease outcomes (e.g., mobility/disability for physical performance biomarkers or cardiovascular-related outcomes for cardiac biomarkers). In conclusion, our findings provide strong support for the continual implementation of routine frailty assessment combining biomarkers in health care settings and advocate caution against trying to capture frailty and mortality risk using a single biomarker.

### Supplementary Information

Below is the link to the electronic supplementary material.

Supplementary file1 (DOCX 29.4 KB)

## Data Availability

Data are available from the Canadian Longitudinal Study on Aging (www.clsa-elcv.ca) for researchers who meet the criteria for access to de-identified CLSA data.
