# Supplementary material for: Prognostic accuracy of 70 individual frailty biomarkers in predicting mortality in the Canadian Longitudinal Study on Aging
Source: GeroScience. 2024 Jan 6;46(3):3061–9. doi: 10.1007/s11357-023-01055-2 (PMC11009196; doi:10.1007/s11357-023-01055-2)
Supplement: Supplementary file 1 — Supplementary file1 (DOCX 29.4 KB) [file 11357_2023_1055_MOESM1_ESM.docx]

**Supplemental File 1**. Baseline characteristics comparing those who survived and those who died

|  | **Survived (n=28442)** | **Died (n= 899)** |
| --- | --- | --- |
| Age (years) | 63 ±10 | 72 ±9 |
| FI- Blood | 0.16±0.19 | 0.24±0.13 |
| FI-Examination | 0.29±0.08 | 0.38±0.10 |
| **Sex, n(%)** |  |  |
| Male | 13860 (49%) | 549 (61%) |
| Female | 14582 (51%) | 350 (39%) |
| **Education, n(%)** |  |  |
| Less than secondary school graduation | 1495 (5%) | 89 (10%) |
| Secondary school graduation | 2662 (9%) | 106 (12%) |
| Some post-secondary | 2063 (7%) | 107 (12%) |
| Post-secondary degree/diploma | 22179 (78%) | 594 (66%) |
| Missing | 43 | 3 |

| **Supplementary File 2.** Area under the receiving operating characteristic (AUC) with 95 % confidence intervals for blood-based biomarkers in predicting mortality across four models | | | | | | |
| --- | --- | --- | --- | --- | --- | --- |
| **Biomarker** | **Sample size (n)** | | **Model 1:**  **Sex & age** | **Model 2:**  **Sex, age & biomarker** | **Model 3:**  **Sex, age & 22-item FI-Blood** | **Model 4:** ^b^  **Sex, age, biomarker & 22-item FI-Blood** |
| 1. Red blood cell distribution width | | 25 253 | 0.762 (0.745, 0.779) | 0.783 (0.767, 0.799) ^a^ | 0.787 (0.770, 0.803) ^a^ | 0.797 (0.780, 0.812) ^b c^ |
| 1. High Sensitivity C-Reactive Protein | | 25 252 | 0.762 (0.745, 0.779) | 0.771 (0.754, 0.788) ^a^ | 0.788 (0.772, 0.805) ^a^ | 0.791 (0.774, 0.807) ^b c^ |
| 1. White blood cells | | 25 253 | 0.762 (0.745, 0.779) | 0.768 (0.751, 0.785) ^a^ | 0.787 (0.770, 0.803) ^a^ | 0.790 (0.773, 0.806) ^b c^ |
| 1. Hemoglobin A1c | | 25 154 | 0.761 (0.744, 0.778) | 0.768 (0.751, 0.785) ^a^ | 0.787 (0.771, 0.804) ^a^ | 0.790 (0.773, 0.806) ^b^ |
| 1. Hematocrit | | 25 253 | 0.762 (0.745, 0.779) | 0.768 (0.751, 0.785) ^a^ | 0.790 (0.773, 0.806) ^a^ | 0.790 (0.773, 0.806) ^b^ |
| 1. Mean corpuscular hemoglobin | | 25 253 | 0.762 (0.745, 0.779) | 0.763 (0.746, 0.780) ^a^ | 0.789 (0.773, 0.806) ^a^ | 0.790 (0.773, 0.806) ^b^ |
| 1. Hemoglobin | | 25 253 | 0.762 (0.745, 0.779) | 0.769 (0.752, 0.786) ^a^ | 0.789 (0.773, 0.806) ^a^ | 0.790 (0.773, 0.806) ^b^ |
| 1. Red blood cells | | 25 253 | 0.762 (0.745, 0.779) | 0.770 (0.753, 0.787) ^a^ | 0.789 (0.773, 0.806) ^a^ | 0.790 (0.774, 0.807) ^b^ |
| 1. Albumin | | 25 253 | 0.762 (0.745, 0.779) | 0.775 (0.758, 0.792) ^a^ | 0.788 (0.772, 0.804) ^a^ | 0.792 (0.775, 0.809) ^b^ |
| 1. Creatinine | | 25 253 | 0.762 (0.745, 0.779) | 0.768 (0.750, 0.785) ^a^ | 0.789 (0.772, 0.805) ^a^ | 0.789 (0.773, 0.806) ^b^ |
| 1. Free thyroxine | | 25 251 | 0.762 (0.745, 0.779) | 0.764 (0.746, 0.780) ^a^ | 0.790 (0.773, 0.806) ^a^ | 0.790 (0.774, 0.807) ^b^ |
| 1. Mean corpuscular volume | | 25 253 | 0.762 (0.745, 0.779) | 0.764 (0.747, 0.781) | 0.789 (0.773, 0.806) ^a^ | 0.791 (0.774, 0.807) ^b^ |
| 1. Mean platelet volume | | 25 253 | 0.762 (0.745, 0.779) | 0.763 (0.746, 0.780) | 0.789 (0.772, 0.805) ^a^ | 0.789 (0.773, 0.806) ^b^ |
| 1. Cholesterol | | 25 253 | 0.762 (0.745, 0.779) | 0.767 (0.750, 0.784) | 0.790 (0.773, 0.806) ^a^ | 0.791 (0.775, 0.807) ^b^ |
| 1. Ferritin | | 25 242 | 0.762 (0.745, 0.779) | 0.763 (0.746, 0.780) | 0.791 (0.775, 0.808) ^a^ | 0.791 (0.775, 0.807) ^b^ |
| 1. Triglycerides | | 25 253 | 0.762 (0.745, 0.779) | 0.763 (0.746, 0.780) | 0.790 (0.773, 0.806) ^a^ | 0.790 (0.773, 0.806) ^b^ |
| 1. Granulocytes | | 25 253 | 0.762 (0.745, 0.779) | 0.764 (0.747, 0.781) | 0.789 (0.773, 0.806) ^a^ | 0.790 (0.774, 0.807) ^b^ |
| 1. Lymphocytes | | 25 253 | 0.762 (0.745, 0.779) | 0.765 (0.748, 0.781) | 0.790 (0.773, 0.806) ^a^ | 0.791 (0.774, 0.807) ^b^ |
| 1. Monocytes | | 25 253 | 0.762 (0.745, 0.779) | 0.763 (0.746, 0.780) | 0.790 (0.773, 0.806) ^a^ | 0.790 (0.773, 0.806) ^b^ |
| 1. Platelets | | 25 253 | 0.762 (0.745, 0.779) | 0.762 (0.745, 0.779) | 0.788 (0.772, 0.805) ^a^ | 0.788 (0.771, 0.804) ^b^ |
| 1. 25-hydroxyvitamin D 25 246 | | | 0.763 (0.746, 0.780) | 0.763 (0.746, 0.780) | 0.789 (0.772, 0.805) ^a^ | 0.788 (0.772, 0.805) ^b^ |
| 1. Estimated Glomerular 25 253 Filtration Rate | | | 0.762 (0.745, 0.779) | 0.764 (0.747, 0.782) | 0.789 (0.772, 0.805) ^a^ | 0.789 (0.772, 0.805) ^b^ |
| 1. Thyroid-Stimulating hormone | | 25 214 | 0.762 (0.745, 0.779) | 0.763 (0.746, 0.780) | 0.789 (0.773, 0.806) ^a^ | 0.790 (0.773, 0.806) ^b^ |
| ^a^ significance denotes improved fit compared to sex & age only model (Model 2 vs Model 1 or Model 3 vs Model 1)  ^b^ significance denotes improved fit compared to sex, age & biomarker model (Model 4 vs Model 3)  ^c^ significance denotes addition of biomarker improves fit compared to sex, age & FI-Blood model (Model 4 v Model 3) | | | | | | |

| **Supplementary File 3.** Area under the receiving operating characteristic (AUC) with 95 % confidence intervals for test-based biomarkers in predicting mortality | | | | | |
| --- | --- | --- | --- | --- | --- |
| **Biomarker** | **Sample size (n)** | **Model 1:**  **Sex & age** | **Model 2:** ^a^  **Sex, age & biomarker** | **Model 3:** ^a^  **Sex, age & 46-item FI- Examination** | **Model 4:**  **Sex, age, biomarker & 46-item**  **FI-Examination** |
| **Physical performance** |  |  |  |  |  |
| 1. Timed 4-metre walk | 29 241 | 0.762 (0.746, 0.778) | 0.776 (0.760, 0.791) ^a^ | 0.785 (0.770, 0.800) ^a^ | 0.790 (0.775, 0.805) ^b^ ^c^ |
| 1. Chair rise | 28 391 | 0.758 (0.741, 0.775) | 0.770 (0.754, 0.787) ^a^ | 0.777 (0.761, 0.794) ^a^ | 0.783 (0.767, 0.799) ^b c^ |
| 1. Timed Get Up & Go | 29 210 | 0.761 (0.745, 0.777) | 0.776 (0.760, 0.791) ^a^ | 0.784 (0.768, 0.799) ^a^ | 0.789 (0.774, 0.804) ^b c^ |
| 1. Standing balance | 28 119 | 0.758 (0.740, 0.775) | 0.770 (0.754, 0.787) ^a^ | 0.775 (0.758, 0.792) ^a^ | 0.781 (0.765, 0.798) ^b^ |
| 1. Grip strength | 27 337 | 0.763 (0.746, 0.779) | 0.771 (0.754, 0.787) ^a^ | 0.786 (0.770, 0.802) ^a^ | 0.790 (0.774, 0.805) ^b^ |
| **Cognition** |  |  |  |  |  |
| 1. Stroop interference time | 29 231 | 0.760 (0.744, 0.776) | 0.766 (0.750, 0.782) ^a^ | 0.785 (0.770, 0.801) ^a^ | 0.787 (0.772, 0.803) ^b^ |
| 1. Delayed recall | 28 389 | 0.761 (0.744, 0.777) | 0.766 (0.750, 0.782) ^a^ | 0.786 (0.770, 0.801) ^a^ | 0.787 (0.771, 0.802) ^b^ |
| 1. Event-based memory | 29 269 | 0.760 (0.744, 0.776) | 0.761 (0.746, 0.777) ^a^ | 0.785 (0.770, 0.800) ^a^ | 0.786 (0.771, 0.801) ^b^ |
| 1. Animal fluency | 29 685 | 0.759 (0.743, 0.775) | 0.762 (0.746, 0.778) ^a^ | 0.785 (0.769, 0.800) ^a^ | 0.785 (0.769, 0.800) ^b^ |
| 1. Controlled Oral Word Association | 28 971 | 0.761 (0.745, 0.776) | 0.761 (0.745, 0.777) | 0.787 (0.772, 0.802) ^a^ | 0.787 (0.772, 0.802) ^b^ |
| 1. Immediate recall | 28 420 | 0.759 (0.743, 0.775) | 0.763 (0.747, 0.779) | 0.784 (0.768, 0.799) ^a^ | 0.784 (0.769, 0.800) ^b^ |
| 1. Mental Alteration Test | 27 959 | 0.763 (0.747, 0.780) | 0.765 (0.748, 0.781) | 0.787 (0.771, 0.802) ^a^ | 0.787 (0.771, 0.802) ^b^ |
| 1. Choice reaction time | 29 109 | 0.760 (0.744, 0.776) | 0.762 (0.746, 0.778) | 0.787 (0.771, 0.802) ^a^ | 0.787 (0.772, 0.802) ^b^ |
| 1. Time-based memory | 29 051 | 0.759 (0.743, 0.775) | 0.760 (0.744, 0.776) | 0.785 (0.770, 0.800) ^a^ | 0.785 (0.770, 0.800) ^b^ |
| **Anthropometric measures** | | | | | |
| 1. Waist-hip ratio | 29 331 | 0.761 (0.745, 0.777) | 0.767 (0.752, 0.782) ^a^ | 0.786 (0.771, 0.801) ^a^ | 0.788 (0.773, 0.803) ^b^ |
| 1. Body mass index | 29 307 | 0.761 (0.746, 0.777) | 0.762 (0.747, 0.778) | 0.787 (0.772, 0.802) ^a^ | 0.787 (0.772, 0.802) ^b^ |
| 1. Whole body bone mineral density, T-score | 28 459 | 0.764 (0.747, 0.779) | 0.763 (0.747, 0.779) | 0.789 (0.774, 0.804) ^a^ | 0.789 (0.774, 0.804) ^b^ |
| 1. Bone mineral density in multiple body regions | 28 459 | 0.764 (0.747, 0.779) | 0.765 (0.749, 0.781) | 0.789 (0.774, 0.804) ^a^ | 0.790 (0.775, 0.805) ^b^ |
| 1. Appendage lean mass | 28 399 | 0.763 (0.747, 0.779) | 0.763 (0.747, 0.779) | 0.788 (0.773, 0.804) ^a^ | 0.790 (0.775, 0.805) ^b^ |
| 1. Body fat percent | 28 473 | 0.764 (0.747, 0.779) | 0.764 (0.748, 0.780) | 0.790 (0.774, 0.805) ^a^ | 0.790 (0.774, 0.805) ^b^ |
| 1. High adiposity in multiple body regions | 28 468 | 0.764 (0.747, 0.779) | 0.765 (0.749, 0.781) | 0.789 (0.774, 0.805) ^a^ | 0.790 (0.774, 0.804) ^b^ |
| **Spirometry** |  |  |  |  |  |
| 1. Forced Vital Capacity (FVC) 22 058 | | 0.756 (0.735, 0.778) | 0.766 (0.745, 0. 787) ^a^ | 0.775 (0.753, 0.796) ^a^ | 0.778 (0.757, 0.799) ^b^ |
| 1. Forced Expiratory Volume 1/FVC Ratio | 22 049 | 0.756 (0.734, 0.778) | 0.759 (0.737, 0.780) | 0.775 (0.754, 0.796) ^a^ | 0.777 (0.756, 0.798) ^b^ |
| **Hearing and vision** |  |  |  |  |  |
| 1. Hearing pure tone average, right | 28 208 | 0.760 (0.744, 0.777) | 0.763 (0.746, 0.779) | 0.785 (0.769, 0.800) ^a^ | 0.785 (0.769, 0.800) ^b^ |
| 1. Hearing pure tone average, left | 29 642 | 0.759 (0.743, 0.775) | 0.760 (0.744, 0.776) | 0.784 (0.769, 0.800) ^a^ | 0.784 (0.769, 0.800) ^b^ |
| 1. Visual acuity, left eye | 28 880 | 0.761 (0.745, 0.777) | 0.762 (0.746, 0.778) | 0.787 (0.772, 0.803) ^a^ | 0.787 (0.772, 0.803) ^b^ |
| 1. Visual acuity, right eye | 28 861 | 0.761 (0.745, 0.777) | 0.762 (0.746, 0.778) | 0.786 (0.771, 0.801) ^a^ | 0.786 (0.770, 0.801) ^b^ |
| 1. Intraocular pressure, right | 27 999 | 0.760 (0.744, 0.777) | 0.760 (0.743, 0.776) | 0.790 (0.774, 0.805) ^a^ | 0.790 (0.774, 0.805) ^b^ |
| 1. Intraocular pressure, left | 28 032 | 0.761 (0.745, 0.777) | 0.761 (0.745, 0.777) | 0.789 (0.773, 0.804) ^a^ | 0.790 (0.775, 0.806) ^b^ |
| 1. Corneal hysteresis, right | 28 018 | 0.761 (0.745, 0.777) | 0.762 (0.745, 0.778) | 0.790 (0.775, 0.806) ^a^ | 0.791 (0.776, 0.806) ^b^ |
| 1. Corneal hysteresis, left | 27 985 | 0.760 (0.744, 0.777) | 0.760 (0.744, 0.777) | 0.790 (0.775, 0.806) ^a^ | 0.791 (0.777, 0.807) ^b^ |
| 1. Mean ocular perfusion pressure | 28 092 | 0.762 (0.745, 0.778) | 0.761 (0.745, 0.777) | 0.790 (0.774, 0.805) ^a^ | 0.790 (0.774, 0.805) ^b^ |
| **Cardiac** |  |  |  |  |  |
| 1. Pulse | 29 229 | 0.762 (0.746, 0.778) | 0.768 (0.753, 0.783) ^a^ | 0.788 (0.773, 0.803) ^a^ | 0.794 (0.779, 0.808) ^b c^ |
| 1. Presence of plaques (max carotid intima thickness) | 26 012 | 0.771 (0.755, 0.788) | 0.773 (0.757, 0.790) ^a^ | 0.795 (0.779, 0.811) ^a^ | 0.795 (0.779, 0.811) ^b^ |
| 1. ECG, QT interval | 29 188 | 0.761 (0.745, 0.776) | 0.769 (0.753, 0.784) ^a^ | 0.785 (0.769, 0.800) ^a^ | 0.788 (0.773, 0.803) ^b^ |
| 1. ECG, PQ interval | 27 823 | 0.752 (0.735, 0.769) | 0.751 (0.734, 0.768) | 0.778 (0.762, 0.794) ^a^ | 0.778 (0.762, 0.794) ^b^ |
| 1. ECG, P axis | 27 901 | 0.752 (0.735, 0.769) | 0.753 (0.736, 0.770) | 0.778 (0.761, 0.794) ^a^ | 0.778 (0.762, 0.794) ^b^ |
| 1. ECG, R axis | 29 185 | 0.761 (0.745, 0.776) | 0.761 (0.745, 0.777) | 0.787 (0.772, 0.802) ^a^ | 0.788 (0773, 0.803) ^b^ |
| 1. ECG, T axis | 29 185 | 0.761 (0.745, 0.776) | 0.762 (0.746, 0.778) | 0.787 (0.772, 0.802) ^a^ | 0.787 (0.772, 0.802) ^b^ |
| 1. ECG, P duration | 27 846 | 0.751 (0.734, 0.768) | 0.750 (0.733, 0.767) | 0.777 (0.760, 0.793) ^a^ | 0.776 (0.760, 0.793) ^b^ |
| 1. ECG, QRS duration | 29 188 | 0.761 (0.745, 0.776) | 0.761 (0.746, 0.777) | 0.786 (0.771, 0.801) ^a^ | 0.786 (0.771, 0.801) ^b^ |
| 1. ECG diagnosis summary | 29 188 | 0.761 (0.745, 0.776) | 0.763 (0.747, 0.779) | 0.787 (0.772, 0.802) ^a^ | 0.787 (0.772, 0.802) ^b^ |
| 1. Systolic BP | 29 231 | 0.762 (0.746, 0.778) | 0.762 (0.746, 0.777) | 0.788 (0.773, 0.803) ^a^ | 0.787 (0.772, 0.802) ^b^ |
| 1. Diastolic BP | 29 231 | 0.762 (0.746, 0.778) | 0.761 (0.746, 0.777) | 0.787 (0.772, 0.802) ^a^ | 0.787 (0.772, 0.802) ^b^ |
| 1. Pulse pressure | 29 231 | 0.762 (0.746, 0.778) | 0.762 (0.746, 0.778) | 0.787 (0.772, 0.802) ^a^ | 0.787 (0.772, 0.802) ^b^ |
| 1. Average carotid intima thickness, right side | 25 785 | 0.773 (0.757, 0.790) | 0.773 (0.757, 0.790) | 0.798 (0.782, 0.813) ^a^ | 0.798 (0.782, 0.813) ^b^ |
| 1. Average carotid intima thickness, left side | 24 456 | 0.772 (0.754, 0.789) | 0.772 (0.755, 0.789) | 0.797 (0.781, 0.813) ^a^ | 0.797 (0.781, 0.814) ^b^ |
| ^a^ significance denotes improved fit compared to sex & age only model (Model 2 vs Model 1 or Model 3 vs Model 1)  ^b^ significance denotes improved fit compared to sex, age & biomarker model (Model 4 vs Model 3)  ^c^ significance denotes addition of biomarker improves fit compared to sex, age & FI-Examination model (Model 4 v Model 3) | | | | | |
